# Supplementary material for: Neural population dynamics in motor cortex are different for reach and grasp
Source: eLife. 2020 Nov 17;9:e58848. doi: 10.7554/eLife.58848 (PMC7688308; doi:10.7554/eLife.58848)
Supplement: Supplementary file 1. [file elife-58848-supp1.docx]

**Table 1.** Datasets and related analyses and figures.

| Dataset | Animal | Array(s) | Task | # of Pooled Recordings | jPCA (Fig. 1) | Decoding (Fig. 2) | Tangling (Fig. 3) |
| --- | --- | --- | --- | --- | --- | --- | --- |
| Dataset 1 | Monkey 1 | Gray Matter | Grasp | 6 | X |  | X |
| Dataset 2 | Monkey 2 | Gray Matter | Grasp | 9 | X |  | X |
| Dataset 3 | Monkey 3 | Utah, FMA | Grasp | 1 |  | X |  |
| Dataset 4 | Monkey 1 | Utah | Grasp | 1 |  | X |  |
| Dataset 5 | Monkey 4 | Utah | Reach | 1 | X | X | X |
| Dataset 6 | Monkey 5 | Utah | Reach | 1 | X | X | X |
